# Associations of metabolic score for insulin resistance with type 2 diabetes, cancer, and all-cause mortality: a meta-analysis of cohort studies

**DOI:** 10.3389/fendo.2026.1884402

**Published:** 2026-08-03

**Authors:** Feng Guo, Hui Liu, Junyi Sun, Jiajing Zhang, Jiawei Wang, Wei Liu, Jiangxue Feng

**Affiliations:** 1 Faculty of Nursing, Hebei University of Chinese Medicine, Shijiazhuang, Hebei, China; 2 The First Affiliated Hospital of Hebei University of Chinese Medicine, Shijiazhuang, Hebei, China

**Keywords:** all-cause mortality, cancer, dose-response analysis, meta-analysis, metabolic score for insulin resistance, type 2 diabetes

## Abstract

**Objective:**

Insulin resistance (IR) constitutes a central pathophysiological mechanism underlying a spectrum of chronic metabolic disorders. The Metabolic Score for Insulin Resistance (METS-IR), which integrates fasting blood glucose (FBG), triglycerides (TG), high-density lipoprotein cholesterol (HDL-C), and body mass index (BMI), serves as a simple, cost-effective surrogate marker for IR. However, its associations with type 2 diabetes mellitus (T2DM), cancer, and all-cause mortality remain to be systematically synthesized. This study aims to systematically evaluate its clinical prognostic value.

**Methods:**

We systematically searched PubMed, EMBASE, and Web of Science for prospective or retrospective cohort studies evaluating the associations of baseline METS-IR with incident T2DM, cancer, or all-cause mortality. Pooled hazard ratios (HRs) and 95% confidence intervals (CIs) were calculated using random-effects models, and linear and nonlinear dose-response meta-analyses were performed. Heterogeneity, robustness, and publication bias were additionally assessed.

**Results:**

A total of 23 cohort studies were included. For T2DM (6 studies), the highest METS-IR category was associated with a substantially elevated risk compared with the lowest category (HR = 4.03, 95% CI: 2.57–6.33), and per 1-standard deviation (1-SD) increment in METS-IR corresponded to a 48% higher risk (HR = 1.48, 95% CI: 1.24–1.77); dose-response analysis revealed a significant nonlinear increasing relationship (*P* for nonlinearity< 0.001). For cancer (5 studies), per 1-SD increment in METS-IR was associated with a 21% higher risk of cancer (HR = 1.21, 95% CI: 1.10–1.34); dose-response analysis indicated a significant nonlinear association (*P* for nonlinearity = 0.042). For all-cause mortality (13 studies), per 1-SD rise in METS-IR was linked to an 8% increased risk of all-cause death (HR = 1.08, 95% CI: 1.03–1.14); dose-response analysis demonstrated an overall linear increasing trend (*P* for linearity = 0.029), with each 20-unit increase in METS-IR associated with an approximately 2.8% higher risk of all-cause mortality (HR = 1.028, 95% CI: 1.004–1.056).

**Conclusions:**

Elevated METS-IR levels are significantly associated with a nonlinear, progressive increase in the risk of incident T2DM. Its associations with cancer and all-cause mortality are weaker and more complex yet still exhibit consistent directional trends. METS-IR represents a convenient, readily applicable tool for early identification of metabolic risk using routine clinical parameters; however, its prognostic value for cancer and all-cause mortality merits further validation in large-scale, long-term follow-up studies.

**Systematic review registration**

PROSPERO, identifier CRD420261293948.

## Introduction

In recent years, the growing prevalence of obesity, physical inactivity, and population aging has escalated the burden of metabolic disorders, challenging global public health (1–3). The Metabolic Score for Insulin Resistance (METS-IR), a central component of metabolic dysregulation, is a hallmark of disrupted homeostasis and serves as a shared pathophysiological basis for accumulating chronic disease risk and adverse outcomes (4, 5). Accordingly, early identification of individuals at high IR-related risk is critical for disease prevention, risk stratification, and long-term health management.

The hyperinsulinemic-euglycemic clamp remains the gold standard for assessing IR; however, its complexity and cost preclude widespread application in large-scale epidemiological studies and routine clinical practice (6). Although the homeostasis model assessment of IR (HOMA-IR) is widely utilized, its reliance on fasting insulin measurements limits accessibility in population-based screening (7). The METS-IR, which integrates fasting blood glucose (FBG), triglycerides (TG), high-density lipoprotein cholesterol (HDL-C), and body mass index (BMI), has emerged as a promising surrogate marker for IR due to its reliance on routine, low-cost clinical parameters (8). Compared with other insulin-independent surrogates such as the triglyceride-glucose (TyG) index, METS-IR additionally incorporates BMI, enabling a more comprehensive reflection of the interplay between adiposity accumulation and metabolic dysregulation (9). Previous studies suggest that METS-IR may outperform conventional surrogate markers in predicting visceral obesity, incident diabetes, and metabolic abnormalities (8, 10).

As the application of METS-IR in epidemiological research has expanded, its potential associations with long-term health outcomes have attracted increasing attention (11, 12). IR, accompanied by hyperinsulinemia, dyslipidemia, chronic low-grade inflammation and oxidative stress, not only directly drives the initiation and progression of type 2 diabetes mellitus (T2DM), but may also contribute to the development of certain malignancies via multiple metabolism- and inflammation-associated pathways, and correlates with elevated long-term mortality risk (12–14). Nevertheless, existing evidence remains inconclusive. Discrepancies are particularly evident in studies examining cancer outcomes (15, 16). For all-cause mortality, some investigations have reported linear associations, whereas others suggest potential nonlinear relationships (17, 18). These observations indicate that the association between METS-IR and adverse health outcomes may be complex, necessitating a systematic synthesis and clarification of the available evidence. Furthermore, most systematic reviews on METS-IR have focused on single outcomes, and comprehensive evaluations across diverse long-term health endpoints remain scarce.

The present meta-analysis aims to comprehensively evaluate the associations of METS-IR with T2DM, cancer, and all-cause mortality, and to further conduct linear and nonlinear dose-response analyses. This work seeks to more fully elucidate the prognostic value of METS-IR across distinct clinical outcomes and provide evidence-based support for early risk identification and population health management using routine metabolic markers.

## Methods

This study followed the Preferred Reporting Items for Systematic Reviews and Meta-Analyses (PRISMA) guidelines (**Supplementary Table 1**) and has been registered in PROSPERO (International Prospective Register of Systematic Reviews; registration number: CRD420261293948).

### Literature search

We comprehensively searched PubMed, EMBASE, and Web of Science up to January 26, 2026. Search terms were constructed as combinations of exposure and outcome variables. The exposures included METS-IR, while the outcomes comprised diabetes mellitus, neoplasms, and all-cause mortality. The detailed search strategy is provided in **Supplementary Table 2**. Two investigators independently conducted literature screening using EndNote software and assessed eligibility by sequentially reviewing titles, abstracts, and full-text articles.

### Inclusion and exclusion criteria

Studies were eligible if they: (1) Recruited adults aged ≥18 years with no prior diagnosis of T2DM and no prevalent cancer at baseline; (2) Explicitly used METS-IR as the exposure, calculated as: $ln\left[\left(2\times FBG\left(mg/dL\right)\right)+TG\left(mg/dL\right)\right]\times BMI\left(kg/m^{2}\right))/\left(ln\left[HDL-C\left(mg/dL\right)\right]\right)$; (3) Reported at least one of the following endpoints: T2DM, cancer, or all-cause mortality; (4) Adopted a prospective or retrospective cohort study design.Exclusion criteria: Studies were excluded if they (1) Recruited children or adolescents; (2) Were systematic reviews, meta-analyses, editorials, letters, conference abstracts, case reports, case-control studies, or cross-sectional studies; (3) Were not published in English or not peer-reviewed; (4) Did not report multivariable-adjusted results; (5) Lacked an abstract or full text; (6) Represented duplicate publications; (7) Used ambiguous exposure definitions or METS-IR calculation formulas inconsistent with the present study.

### Data collection

Two researchers independently extracted the following information from each included study: first author, publication year, study population characteristics, study design, follow-up duration, baseline age, sample size, sex distribution, risk estimation methods, covariate adjustments, and effect estimates with 95% CIs. All data were derived from the most extensively adjusted model reported in each study. After extraction, the two researchers cross-checked the data for consistency. Disagreements were resolved by a third researcher, whose decision was final.

### Assessment of study quality and certainty of evidence

The Newcastle-Ottawa Scale (NOS) was used to assess the quality of the included studies. The scale evaluates cohort studies across three domains: participant selection, comparability of groups, and outcome assessment (score range: 1–9 points).

### Statistical analysis

All statistical analyses were performed using Stata 18.0 (StataCorp LLC, Texas, USA) and R 4.4.3 (R Core Team, Vienna, Austria). HRs with corresponding 95% CIs were used as the pooled effect sizes to evaluate associations between baseline METS-IR and T2DM, cancer, and all-cause mortality. For studies treating METS-IR as a categorical variable, HRs comparing the highest versus lowest exposure categories were extracted; for studies treating METS-IR as a continuous variable, HRs per 1-SD increment were retrieved.Specifically, for studies that reported HRs, the HR per 1-SD increment was derived by exponentiating the product of log(HR) and the SD of METS-IR in the respective study.Data were pooled using a random-effects model, and between-study heterogeneity was assessed using the I² statistic and Cochran’s Q test. Statistical significance was set at *P* < 0.05, and significant heterogeneity was defined as I² > 50% or *P* < 0.10 for Cochran’s Q test. Subgroup analyses stratified by study region, proportion of male participants, follow-up duration, sample size, and mean age were conducted to explore potential sources of heterogeneity. A leave-one-out sensitivity analysis was performed to evaluate the robustness of the pooled effect estimates. Publication bias was assessed using Egger’s test (*P* < 0.05 indicating potential bias); if detected, the trim-and-fill method was applied for correction to validate the robustness of the results.

We also extracted categorical dose-response data for inclusion in linear and nonlinear dose-response analyses, respectively. According to the method proposed by Greenland and Longnecker (19), linear trends with 95% CIs were estimated using a one-stage approach (20) based on natural logarithm-transformed effect sizes and their confidence intervals across METS-IR dose categories. Nonlinear dose-response trends were modeled using restricted cubic spline functions with three fixed knots at the 10th, 50th, and 90th percentiles, combined with the one-stage method (20). The goodness of fit of linear and nonlinear models was evaluated using the Akaike Information Criterion (AIC) and log-likelihood values, with a lower AIC and higher log-likelihood indicating better fit (21). For METS-IR reported in categories, the midpoint of each closed interval was assigned as the exposure value; for open-ended intervals, the midpoint was estimated based on the width of the adjacent interval.

## Results

### Literature screening process and results

**Figure 1:**
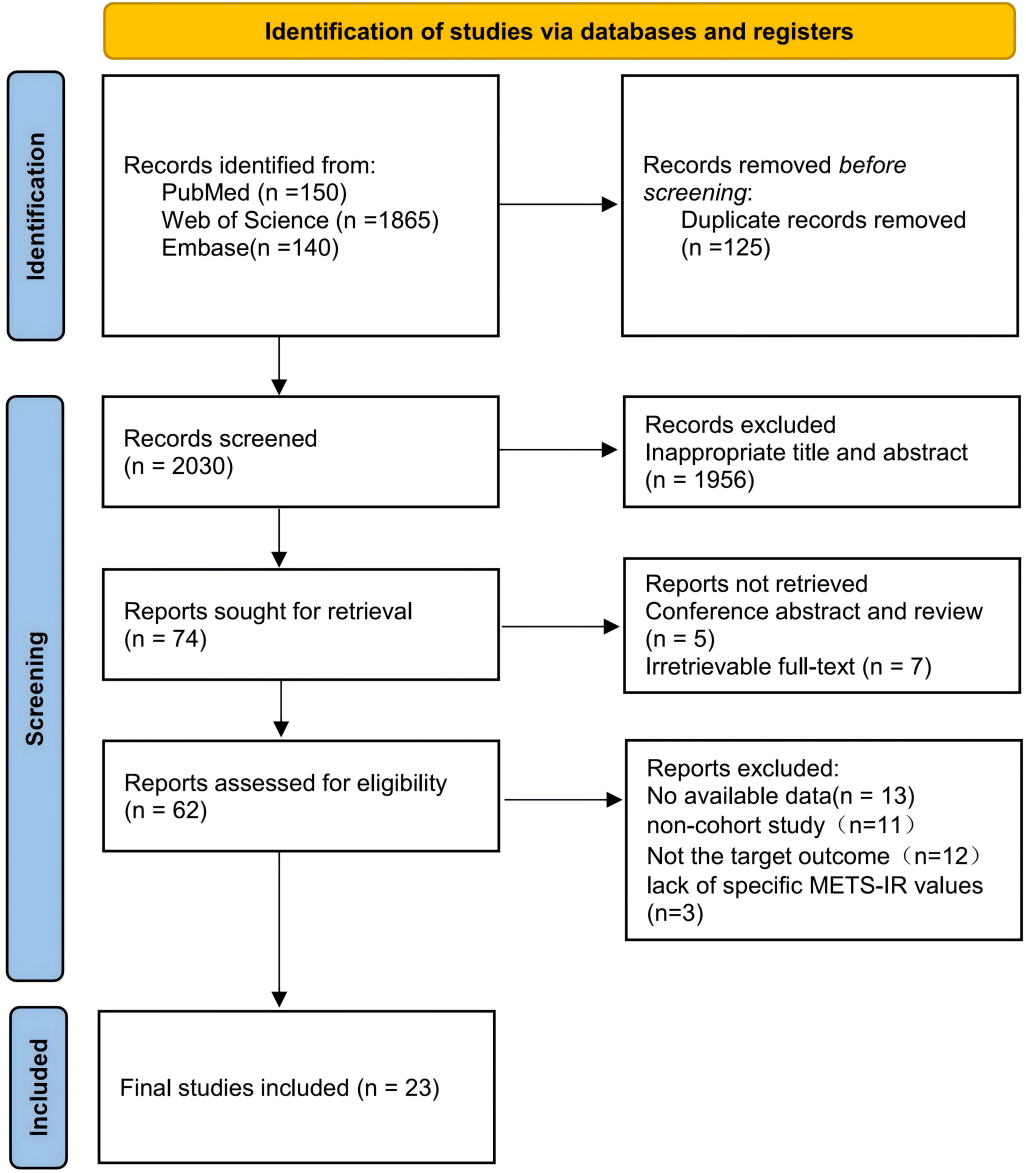
Database search and study screening flowchart.

A total of 2,155 relevant records were identified through searches of PubMed, EMBASE, and Web of Science. After removal of duplicates in EndNote, 2,030 records remained. Title and abstract screening excluded 1,956 irrelevant records; an additional 5 conference abstracts/reviews and 7 records with inaccessible full texts were further removed. Full-text review of the remaining 62 articles led to the exclusion of 39 studies (primary exclusion reasons are provided in **Supplementary Table 3**), yielding 23 studies for inclusion in the meta-analysis (**Figure 1**).

### Baseline characteristics of included studies

Detailed baseline characteristics of the 23 included studies are presented in **Supplementary Table 4**. Of these, 21 were prospective cohort studies (8, 12, 15–18, 22–36)and 2 were retrospectivecohort studies (37, 38), with geographic distribution as follows: 11 studies (15, 29, 32, 33, 35–37)from Asia (primarily China, South Korea, Japan, and Iran), 3 studies (16, 26, 28)from Europe (United Kingdom), 8 studies (12, 18, 22–25, 31, 34)from North America (United States), and 1 studies (8)from South America (Mexico). Six studies examined T2DM as the primary outcome (total n = 480,250; mean age range, 42.63–57.8 years; male proportion, 33.4%–53.9%; follow-up, 2.42–13.84 years). Five studies investigated cancer (total n = 1,735,290; mean age range, 56.3–58.8 years; male, 46.4%–54.48%; follow-up, 9.5–13 years). Thirteen studies assessed all-cause mortality (total n = 417,650; mean age range, 41.7–65.7 years; male, 42.6%–59%; follow-up, 3.94–23.3 years).

### Quality assessment of included studies

We assessed study quality using the NOS (maximum score, 9 points). Ten studies scored 9 points and 13 scored 8 points, indicating overall high methodological quality among the included cohort studies. Detailed assessment results are presented in **Supplementary Table 5**.

## Results of the meta-analysis

### Association of METS-IR with the risk of incident T2DM

**Figure 2:**
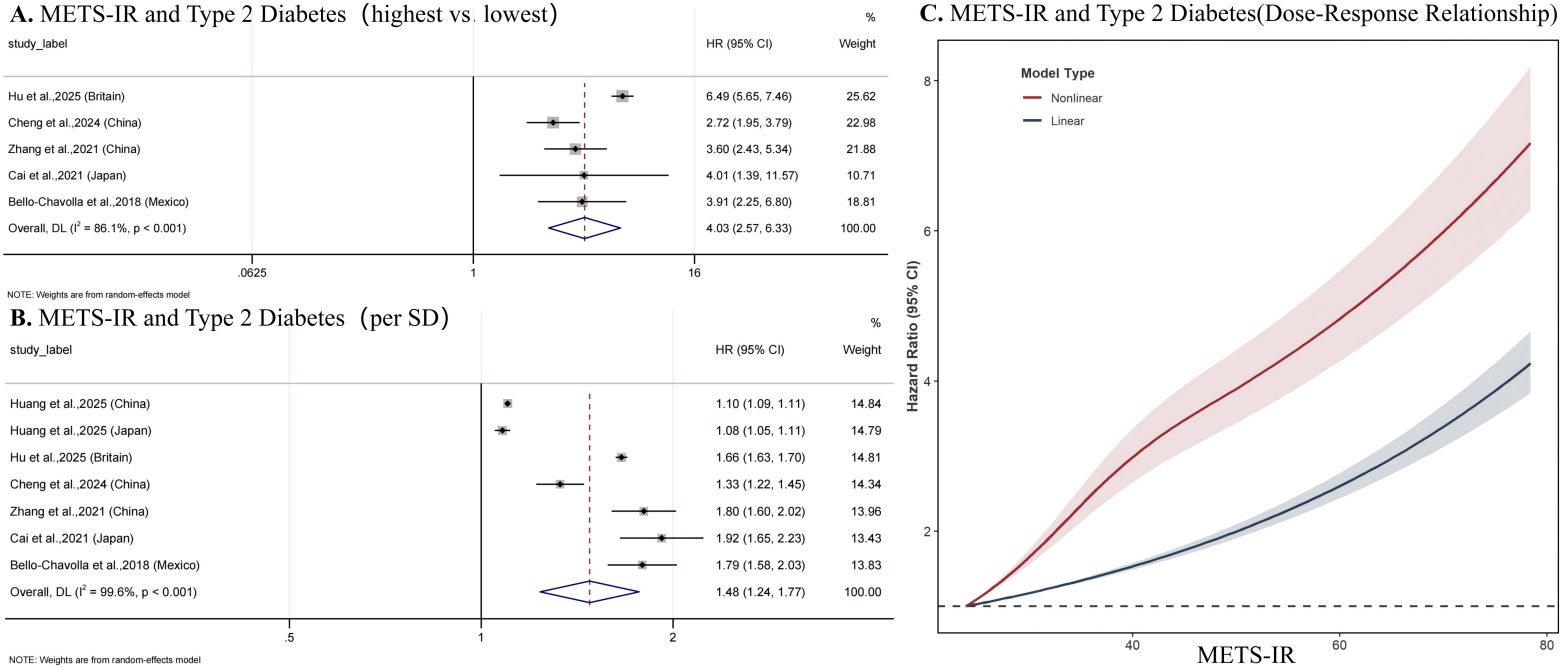
Meta-analysis results of METS-IR and T2DM. **(A)** METS-IP and type 2 diabetes (highest vs. lowest). **(B)** METS-IP and type 2 diabetes (per SD). **(C)** METS-IP and type 2 diabetes (dose-response relationship).

Six cohort studies evaluated the association of METS-IR with incident T2DM. Categorical analysis revealed that participants in the highest METS-IR category had a significantly higher risk of T2DM than those in the lowest category (HR = 4.03, 95% CI: 2.57–6.33, I² = 86.1%) (**Figure 2A**). Per 1-SD increment in METS-IR, the risk of incident T2DM increased by 48% (HR = 1.48, 95% CI: 1.24–1.77, I² = 99.6%) (**Figure 2B**). Dose-response analysis indicated a significant nonlinear association between METS-IR and T2DM risk (N = 5, *P* for nonlinearity< 0.001, AIC = 63.74, log-likelihood = −29.87; *P* for linearity< 0.001, AIC = 191.97, log-likelihood = −94.98) (**Figure 2C**). The risk of incident T2DM increased nonlinearly with rising METS-IR levels.

### Association of METS-IR with the risk of incident cancer

**Figure 3:**
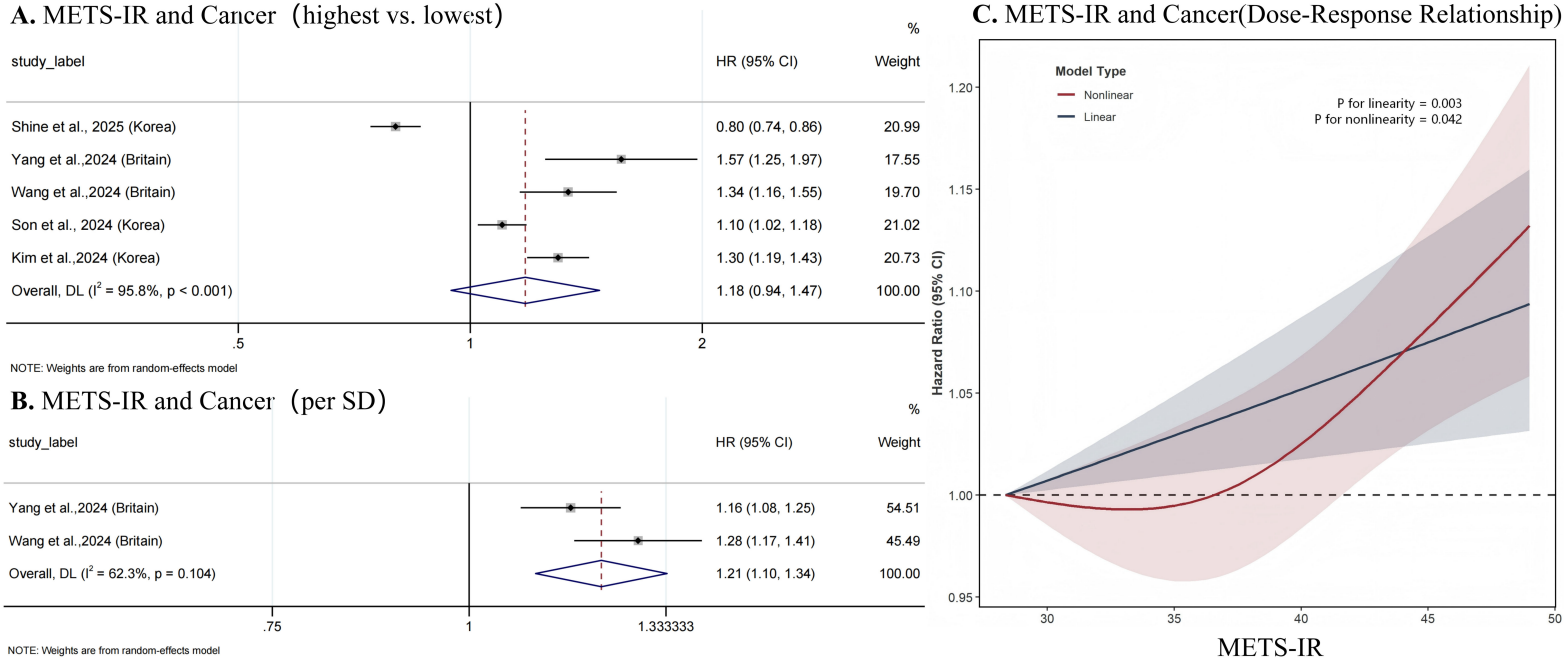
Meta-analysis results of METS-IR and Cancer. **(A)** METS-IR and cancer (highest vs. lowest). **(B)** METS-IR and cancer (per SD). **(C)** METS-IR and cancer (dose-response relationship).

Five cohort studies examined the association of METS-IR with incident cancer. Categorical analysis showed no significant difference in cancer risk between the highest and lowest METS-IR categories (HR = 1.18, 95% CI: 0.94–1.47, I² = 95.8%) (**Figure 3A**). Per 1-SD increment in METS-IR, cancer risk increased by 21% (HR = 1.21, 95% CI: 1.10–1.34, I² = 62.3%) (**Figure 3B**). Dose-response analysis revealed a nonlinear association between METS-IR and incident cancer risk (N = 5, *P* for nonlinearity = 0.042, AIC = 45.64, log-likelihood = −20.82; *P* for linearity = 0.003, AIC = 47.78, log-likelihood = −22.89) (**Figure 3C**), with a more pronounced increase in risk at higher METS-IR levels.

### Association of METS-IR with all-cause mortality

**Figure 4:**
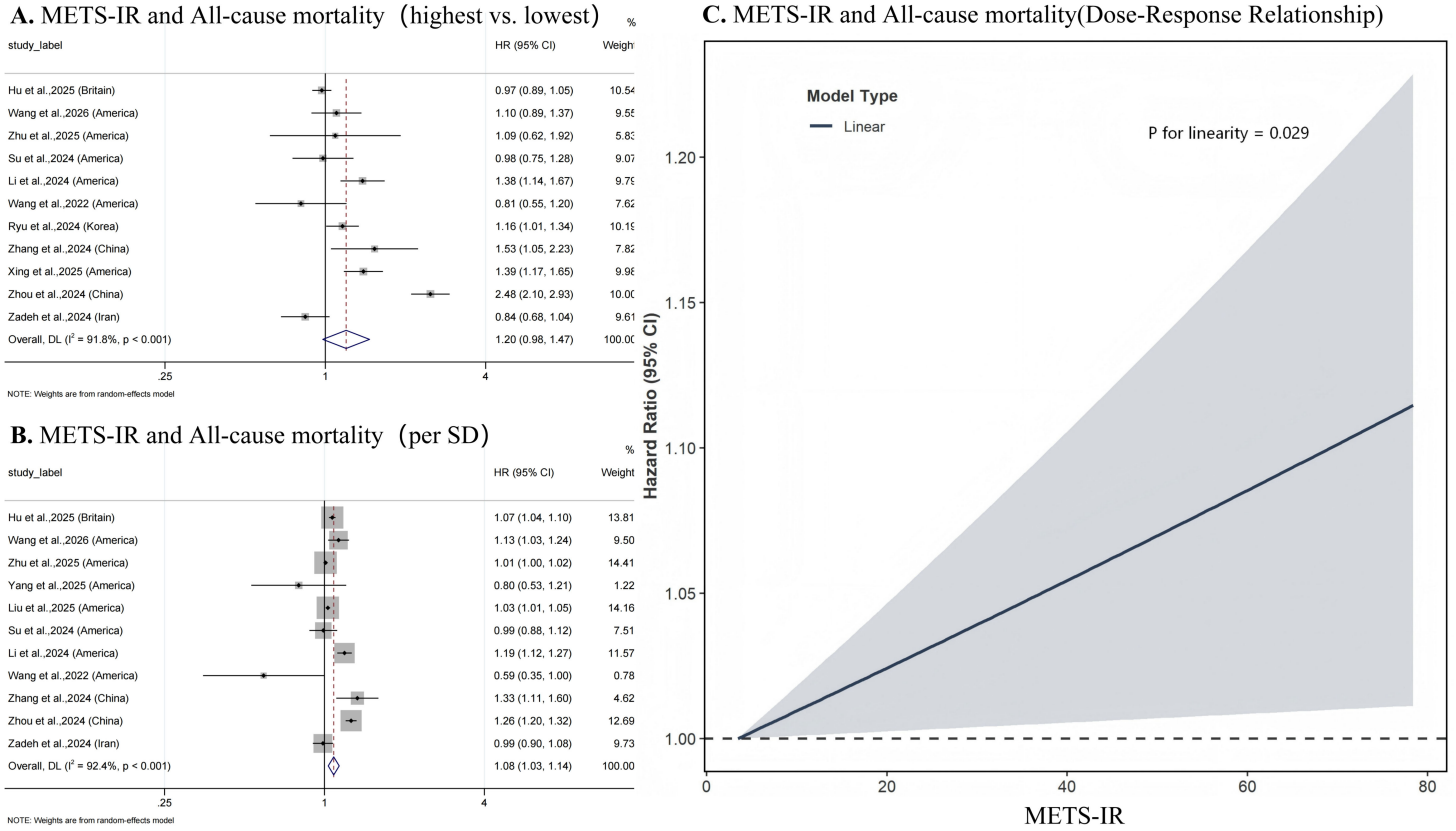
Meta-analysis results of METS-IR and All-cause mortality. **(A)** METS-IR and all-cause mortality (highest vs. lowest). **(B)** METS-IR and all-cause mortality (per SD). **(C)** METS-IR and all-cause mortality (dose-response relationship).

Thirteen cohort studies examined the association of METS-IR with all-cause mortality. Categorical analysis showed no significant difference in all-cause mortality between the highest and lowest METS-IR categories (HR = 1.20, 95% CI: 0.98–1.47, I² = 95.8%) (**Figure 4A**). Per 1-SD increment in METS-IR, all-cause mortality risk increased by 8% (HR = 1.08, 95% CI: 1.03–1.14, I² = 62.3%) (**Figure 4B**). Dose-response analysis indicated a linear association between METS-IR and all-cause mortality (N = 11, *P* for linearity = 0.029, AIC = 108.74, log-likelihood = −53.37; *P* for nonlinearity = 0.081, AIC = 107.69, log-likelihood = −51.85) (**Figure 4C**). Each 20-unit increment in METS-IR was associated with an approximately 2.8% higher risk of all-cause mortality (HR = 1.028, 95% CI: 1.004–1.056).

### Subgroup analyses

**Figure 5:**
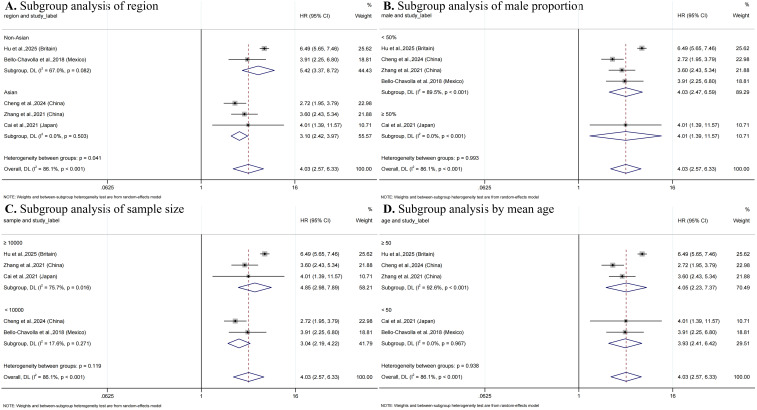
Subgroup analysis of METS-IR (highest vs. lowest) and T2DM. **(A)** Subgroup analysis of region. **(B)** Subgroup analysis of male proportion. **(C)** Subgroup analysis of simple size. **(D)** Subgroup analysis by mean age.

**Figure 6:**
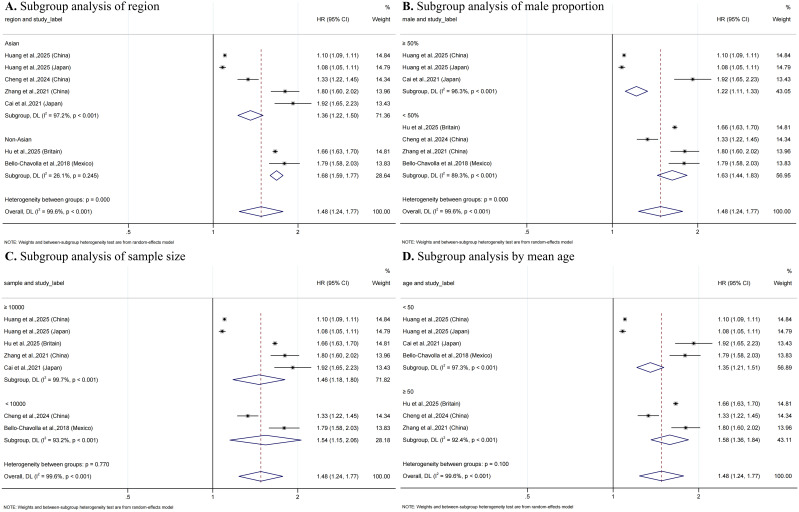
Subgroup analysis of METS-IR (per SD) and T2DM. **(A)** Subgroup analysis of region. **(B)** Subgroup analysis of male proportion. **(C)** Subgroup analysis of simple size. **(D)** Subgroup analysis by mean age.

To explore sources of heterogeneity, we conducted subgroup analyses for T2DM, cancer, and all-cause mortality. For T2DM, analyses were stratified by study region, proportion of male participants, sample size, and mean age. In categorical METS-IR analysis, significant differences emerged across geographic regions (all *P* for interaction< 0.05), while no interactions were observed for the remaining indices (all *P* for interaction > 0.05) (**Figure 5**). Continuous analysis (per 1-SD increment) revealed significant interactions by study region and male proportion (all *P* for interaction< 0.05), with no heterogeneity detected for other indices (all *P* for interaction > 0.05) (**Figure 6**).

For the cancer outcome, subgroup analyses of categorical METS-IR estimates were performed stratified by study region, proportion of male participants, and follow-up duration (**Supplementary Figure 1**), and no significant heterogeneity was detected. Subgroup analysis was not performed for continuous METS-IR (per 1-SD) owing to the limited number of studies (n = 2).

For all-cause mortality, subgroup analyses were stratified by study region, proportion of male participants, sample size, and mean age. Categorical METS-IR subgroup analysis revealed no significant heterogeneity, and none of the subgroup-specific results were statistically significant (**Supplementary Figure 2**). Parallel subgroup analyses of continuous METS-IR estimates similarly identified no significant sources of heterogeneity. Notably, the association between METS-IR and all-cause mortality was not statistically significant in subgroups with a sample size<10,000, male proportion ≥50%, or mean participant age<50 years (**Supplementary Figure 3**).

### Sensitivity analysis and publication bias

Leave-one-out sensitivity analysis was conducted to assess the robustness of pooled effect estimates; all results are presented in **Supplementary Figure 4**. For T2DM, both categorical and continuous METS-IR pooled estimates remained robust, with no change in effect direction or statistical significance. For cancer, sensitivity analysis of categorical METS-IR showed that exclusion of Shine et al. (15) altered the pooled estimate from non-significant to significant, whereas removal of any other single study did not materially change the results, suggesting limited robustness. For all-cause mortality, categorical METS-IR results remained statistically non-significant after sequential exclusion of individual studies, indicating stable findings; continuous METS-IR estimates were robust. Publication bias was assessed using Egger’s test for all analytical indicators. All *P*-values were >0.05, indicating no evidence of publication bias among the included studies (**Supplementary Figure 5**).

## Discussion

This study systematically synthesized 23 cohort studies to comprehensively evaluate the associations of METS-IR with T2DM, cancer, and all-cause mortality. The results demonstrated that higher METS-IR levels were significantly associated with an elevated risk of incident T2DM, and dose-response analysis indicated a significant nonlinear increasing relationship. For cancer, categorical analysis showed no significant association, whereas continuous analysis revealed that elevated METS-IR was linked to increased cancer risk, with dose-response analysis further suggesting a potential nonlinear relationship. For all-cause mortality, no significant association was observed in categorical analysis, while continuous analysis indicated that increased METS-IR was associated with higher all-cause mortality risk. Dose-response analysis supported an overall linear increasing trend, with each 20-unit increment in METS-IR corresponding to an approximately 2.8% rise in all-cause mortality risk. These findings indicate that the prognostic utility of METS-IR may hinge on the magnitude of pathological associations between distinct health outcomes and IR-related metabolic derangements.

METS-IR showed a strong, consistent association with incident T2DM, consistent with the established role of IR in diabetes pathogenesis (39). IR is a core mechanism driving the onset and progression of T2DM, as declining systemic insulin sensitivity initially prompts pancreatic β-cells to maintain glucose homeostasis through compensatory hypersecretion, yet progressive failure of this response ultimately impairs glucose metabolism and promotes diabetes development (40, 41). By incorporating FBG, TG, BMI, and HDL-C, METS-IR captures the combined burden of dysglycemia, dyslipidemia, and obesity-related metabolic dysfunction (8). Notably, elevated TG and reduced HDL-C typically reflect lipotoxicity and dysregulated lipid metabolism, whereas BMI partly reflects whole-body adiposity and associated IR (42–44). Accordingly, METS-IR is more likely than individual metabolic markers to capture the global metabolic imbalance preceding diabetes onset. Furthermore, dose-response analysis revealed a significant nonlinear relationship, indicating that diabetes risk accelerates at higher METS-IR levels rather than increasing uniformly. Despite substantial between-study heterogeneity, subgroup analyses revealed that study region and proportion of male participants could partially account for such discrepancies, and sensitivity analyses verified the robustness of our outcomes. These observations suggest that METS-IR may facilitate early identification and risk stratification of individuals at high risk for T2DM, yet its applicability across diverse populations and optimal clinical cutoffs warrant further prospective validation.For cancer, categorical analysis revealed no significant association, whereas continuous analysis linked higher METS-IR to increased risk. This discrepancy likely reflects the substantial heterogeneity inherent in cancer outcomes. Notably, cancers do not constitute a homogeneous single outcome; malignancies arising from distinct anatomical sites exhibit marked disparities in metabolic reliance, inflammatory microenvironments, hormonal milieu, and obesity-driven mechanisms, and pooled analyses may attenuate genuine associations (29, 45). Furthermore, the pool of eligible studies remained limited: only five studies were included for categorical variable analyses and merely two for continuous variable analyses, which may compromise the stability of pooled estimates. In sensitivity analysis, exclusion of Shine et al. (15) shifted the categorical result from non-significant to significant, further underscoring the limited robustness of these findings. Accordingly, current evidence is insufficient to support definitive conclusions regarding the association between METS-IR and cancer risk, and these results warrant cautious interpretation. Nonetheless, dose-response analysis suggested a potential nonlinear relationship, with cancer risk rising more steeply at higher METS-IR levels. From a biological perspective, IR may promote tumorigenesis and progression through hyperinsulinemia, activation of insulin-like growth factor signaling, chronic low-grade inflammation, and oxidative stress, providing a plausible mechanistic basis for the observed association (46, 47).

All-cause mortality, as a long-term composite endpoint, is influenced not only by metabolic dysfunction but also by age, comorbidities, lifestyle, socioeconomic status, and healthcare access, yielding a complex risk profile (48–51). Prior studies of METS-IR and all-cause mortality have yielded inconsistent findings. For instance, METS-IR exhibited a nonlinear association with mortality in U.S. adults with diabetes (34), a linear relationship in Chinese hypertensive patients (17), and a nonlinear pattern in an Iranian cohort (27). Nevertheless, our dose-response analysis revealed an overall linear increasing trend, with each 20-unit increment in METS-IR corresponding to an approximately 2.8% higher mortality risk—indicating a modest cumulative effect. These discrepancies likely reflect variation in comorbidity burden and population characteristics across studies. Furthermore, although subgroup analyses identified no clear sources of heterogeneity, the continuous association was not statistically significant in subgroups with sample size<10,000, male proportion ≥50%, or mean age<50 years, further suggesting that the METS-IR–mortality relationship may be influenced by sample characteristics and population structure.

Comparison across outcomes reveals hierarchical variation in the associations of METS-IR with distinct health endpoints. These disparities likely reflect the distinct roles of IR in different pathogenic cascades. In T2DM, IR is a direct proximal mechanism (39); for cancer and all-cause mortality, its effects are indirect—mediated through metabolic inflammation, obesity, dyslipidemia, and organ damage—rendering associations more vulnerable to confounding (52, 53). Meanwhile, categorical analyses, continuous variable analyses, and dose–response analyses adopt distinct approaches for exposure processing, which may also yield inconsistent findings. Categorical analyses are heavily influenced by study-specific cutoffs for METS-IR stratification, whereas continuous and dose–response analyses deliver richer information on exposure gradients; accordingly, discrepancies across analytical outcomes may mirror the intricate nature of exposure–outcome relationships. In addition, disparities in study geographic regions, participant profiles, follow-up durations, and covariate adjustment strategies may collectively contribute to the observed inconsistencies and heterogeneity.

This meta-analysis synthesized cohort evidence regarding associations between METS-IR and multiple endpoints, including T2DM, cancer, and all-cause mortality. By integrating categorical, continuous, and dose–response analyses, we comprehensively evaluated the prognostic performance and applicable scope of METS-IR. Several limitations of this work should be acknowledged. First, all included studies were observational; although most implemented multivariable adjustment, residual confounding and reverse causality could not be entirely ruled out. Second, only English-language publications were retrieved while non-English literature was excluded, introducing potential risks of publication bias. Third, the number of cancer-focused studies was limited, with inconsistent cancer definitions and uneven coverage of anatomical sites, restricting subgroup exploration and the generalizability of pooled results. Intrinsic disparities may exist in the links between distinct cancer subtypes and insulin resistance, and pooled syntheses may dilute subtype-specific effect sizes. Moreover, study weights were allocated according to statistical variance rather than balanced distribution across cancer categories. Hence, cancer-related findings should be interpreted as exploratory summaries of overall malignancy risk and require rigorous validation via additional subtype-specific investigations. Fourth, substantial statistical heterogeneity was detected in several analyses, which may undermine the precision and external validity of pooled estimates. Subgroup and sensitivity analyses confirmed the overall stability of core outcomes, yet inter-study discrepancies remained incompletely elucidated, mandating cautious interpretation of our observations. Lastly, all-cause mortality represents a multifactorial composite endpoint. Its correlation with METS-IR can be modulated by age, comorbidities, lifestyle patterns, healthcare access and other confounders, leaving limited capacity for mechanistic inference. Large-scale, long-term, endpoint-specific prospective studies are warranted in future work to further validate the predictive capacity and clinical applicability of METS-IR.

## Conclusions

In summary, this meta-analysis demonstrates that higher METS-IR levels are associated with a significantly elevated, nonlinear risk of T2DM. Associations with cancer and all-cause mortality are weaker and more complex, though directionally consistent. As a simple score derived from routine clinical parameters, METS-IR offers a practical tool for population-level identification of individuals at elevated metabolic risk. Large-scale, long-term prospective studies are warranted to validate its utility across diverse populations, specific cancer types, and mortality risk strata, and to define clinically meaningful risk thresholds.

## Data Availability

The original contributions presented in the study are included in the article/**Supplementary Material**. Further inquiries can be directed to the corresponding author.
