# Supplementary material for: Associations of metabolic score for insulin resistance with type 2 diabetes, cancer, and all-cause mortality: a meta-analysis of cohort studies
Source: Front Endocrinol (Lausanne). 2026 Aug 3;17:1884402. doi: 10.3389/fendo.2026.1884402 (PMC13478956; doi:10.3389/fendo.2026.1884402)
Supplement: Supplementary file 1 [file Table1.docx]

**Supplementary Table S1** PRISMA 2020 Checklist

| **Section and Topic** | **Item #** | **Checklist item** | **Location where item is reported** |
| --- | --- | --- | --- |
| **TITLE** | | |  |
| Title | 1 | Identify the report as a systematic review. | Title |
| **ABSTRACT** | | |  |
| Abstract | 2 | See the PRISMA 2020 for Abstracts checklist. | Abstract |
| **INTRODUCTION** | | |  |
| Rationale | 3 | Describe the rationale for the review in the context of existing knowledge. | introduction |
| Objectives | 4 | Provide an explicit statement of the objective(s) or question(s) the review addresses. | introduction |
| **METHODS** | | |  |
| Eligibility criteria | 5 | Specify the inclusion and exclusion criteria for the review and how studies were grouped for the syntheses. | Inclusion and Exclusion Criteria |
| Information sources | 6 | Specify all databases, registers, websites, organisations, reference lists and other sources searched or consulted to identify studies. Specify the date when each source was last searched or consulted. | Literature search |
| Search strategy | 7 | Present the full search strategies for all databases, registers and websites, including any filters and limits used. | Literature search |
| Selection process | 8 | Specify the methods used to decide whether a study met the inclusion criteria of the review, including how many reviewers screened each record and each report retrieved, whether they worked independently, and if applicable, details of automation tools used in the process. | Literature search |
| Data collection process | 9 | Specify the methods used to collect data from reports, including how many reviewers collected data from each report, whether they worked independently, any processes for obtaining or confirming data from study investigators, and if applicable, details of automation tools used in the process. | Data Collection |
| Data items | 10a | List and define all outcomes for which data were sought. Specify whether all results that were compatible with each outcome domain in each study were sought (e.g. for all measures, time points, analyses), and if not, the methods used to decide which results to collect. | Data Collection |
|  | 10b | List and define all other variables for which data were sought (e.g. participant and intervention characteristics, funding sources). Describe any assumptions made about any missing or unclear information. | Data Collection |
| Study risk of bias assessment | 11 | Specify the methods used to assess risk of bias in the included studies, including details of the tool(s) used, how many reviewers assessed each study and whether they worked independently, and if applicable, details of automation tools used in the process. | Assessment of Study Quality and Certainty of Evidence |
| Effect measures | 12 | Specify for each outcome the effect measure(s) (e.g. risk ratio, mean difference) used in the synthesis or presentation of results. | Statistical Analysis |
| Synthesis methods | 13a | Describe the processes used to decide which studies were eligible for each synthesis (e.g. tabulating the study intervention characteristics and comparing against the planned groups for each synthesis (item #5)). | Statistical Analysis |
|  | 13b | Describe any methods required to prepare the data for presentation or synthesis, such as handling of missing summary statistics, or data conversions. | Statistical Analysis |
|  | 13c | Describe any methods used to tabulate or visually display results of individual studies and syntheses. | Statistical Analysis |
|  | 13d | Describe any methods used to synthesize results and provide a rationale for the choice(s). If meta-analysis was performed, describe the model(s), method(s) to identify the presence and extent of statistical heterogeneity, and software package(s) used. | Statistical Analysis |
|  | 13e | Describe any methods used to explore possible causes of heterogeneity among study results (e.g. subgroup analysis, meta-regression). | Statistical Analysis |
|  | 13f | Describe any sensitivity analyses conducted to assess robustness of the synthesized results. | Statistical Analysis |
| Reporting bias assessment | 14 | Describe any methods used to assess risk of bias due to missing results in a synthesis (arising from reporting biases). | Statistical Analysis |
| Certainty assessment | 15 | Describe any methods used to assess certainty (or confidence) in the body of evidence for an outcome. | N/A |
| **RESULTS** | | |  |
| Study selection | 16a | Describe the results of the search and selection process, from the number of records identified in the search to the number of studies included in the review, ideally using a flow diagram. | Literature Screening Process and Results |
|  | 16b | Cite studies that might appear to meet the inclusion criteria, but which were excluded, and explain why they were excluded. | Literature Screening Process and Results |
| Study characteristics | 17 | Cite each included study and present its characteristics. | Baseline Characteristics of Included Studies |
| Risk of bias in studies | 18 | Present assessments of risk of bias for each included study. | Supplementary Table S5 |
| Results of individual studies | 19 | For all outcomes, present, for each study: (a) summary statistics for each group (where appropriate) and (b) an effect estimate and its precision (e.g. confidence/credible interval), ideally using structured tables or plots. | Results of the Meta-Analysis |
| Results of syntheses | 20a | For each synthesis, briefly summarise the characteristics and risk of bias among contributing studies. | Results of the Meta-Analysis |
|  | 20b | Present results of all statistical syntheses conducted. If meta-analysis was done, present for each the summary estimate and its precision (e.g. confidence/credible interval) and measures of statistical heterogeneity. If comparing groups, describe the direction of the effect. | Results of the Meta-Analysis |
|  | 20c | Present results of all investigations of possible causes of heterogeneity among study results. | Results of the Meta-Analysis |
|  | 20d | Present results of all sensitivity analyses conducted to assess the robustness of the synthesized results. | Results of the Meta-Analysis |
| Reporting biases | 21 | Present assessments of risk of bias due to missing results (arising from reporting biases) for each synthesis assessed. | N/A |
| Certainty of evidence | 22 | Present assessments of certainty (or confidence) in the body of evidence for each outcome assessed. | N/A |
| **DISCUSSION** | | |  |
| Discussion | 23a | Provide a general interpretation of the results in the context of other evidence. | Discussion |
|  | 23b | Discuss any limitations of the evidence included in the review. | Discussion |
|  | 23c | Discuss any limitations of the review processes used. | Discussion |
|  | 23d | Discuss implications of the results for practice, policy, and future research. | Discussion |
| **OTHER INFORMATION** | | |  |
| Registration and protocol | 24a | Provide registration information for the review, including register name and registration number, or state that the review was not registered. | Methods |
|  | 24b | Indicate where the review protocol can be accessed, or state that a protocol was not prepared. | Methods |
|  | 24c | Describe and explain any amendments to information provided at registration or in the protocol. | N/A |
| Support | 25 | Describe sources of financial or non-financial support for the review, and the role of the funders or sponsors in the review. | Funding |
| Competing interests | 26 | Declare any competing interests of review authors. | Conflict of interest |
| Availability of data, code and other materials | 27 | Report which of the following are publicly available and where they can be found: template data collection forms; data extracted from included studies; data used for all analyses; analytic code; any other materials used in the review. | Data Availability |

^a^Locations were based on the submitted manuscript file.

**N/A**, not applicable or not available; **PRISMA**, Preferred Reporting Items for Systematic Reviews and Meta-Analyses.

**Supplementary Table S2** Search strategies

| PubMed | |
| --- | --- |
| #1 | (Metabolic score for insulin resistance[Title/Abstract]) OR (METS-IR[Title/Abstract]) |
| #2 | (((("Diabetes Mellitus"[Mesh]) OR (Diabetes Mellitus[Title/Abstract])) OR (type 2 diabetes mellitus[Title/Abstract])) OR (T2DM[Title/Abstract])) OR (non-insulin-dependent diabetes mellitus[Title/Abstract]) |
| #3 | (((("Neoplasms"[Mesh]) OR (Neoplasms[Title/Abstract])) OR (Tumors[Title/Abstract])) OR (Cancer[Title/Abstract])) OR (Malignancy[Title/Abstract]) |
| #4 | ((mortality[Title/Abstract]) OR (Death[Title/Abstract])) OR (all-cause mortality[Title/Abstract]) |
| #5 | #2 OR #3 OR #4 |
| #6 | #1 AND #5 |
| Embase | |
| #1 | 'metabolic score for insulin resistance':ab,ti OR 'mets ir':ab,ti |
| #2 | 'diabetes mellitus':ab,ti OR 'type 2 diabetes mellitus':ab,ti OR t2dm:ab,ti OR 'non insulin dependent diabetes mellitus':ab,ti |
| #3 | 'neoplasm':ab,ti OR tumors:ab,ti OR cancer:ab,ti OR 'malignancy':ab,ti |
| #4 | 'mortality':ab,ti OR 'all cause mortality':ab,ti OR 'death':ab,ti |
| #5 | #2 OR #3 OR #4 |
| #6 | #1 AND #5 |
| Web of Science | |
| #1 | Metabolic score for insulin resistance (Topic) or METS-IR (Topic) |
| #2 | Diabetes Mellitus (Topic) or type 2 diabetes mellitus (Topic) or T2DM (Topic) or non-insulin-dependent diabetes mellitus (Topic) |
| #3 | Neoplasms (Topic) or Tumors (Topic) or Cancer (Topic) or Malignancy (Topic) |
| #4 | mortality (Topic) or all-cause mortality (Topic) or Death (Topic) |
| #5 | #2 OR #3 OR #4 |
| #6 | #1 AND #5 |

**Supplementary Table S3** List of excluded studies, with reasons for exclusion after full text reading.

|  | References | Reason for exclusion |
| --- | --- | --- |
| 1 | Comparing the effectiveness of different indicators of insulin resistance in predicting diabetes among adults with sarcopenia from the NHANES (1999-2018) | non-cohort study |
| 2 | Sex-specific optimal cut-off points for metabolic health indicators to predict incident type 2 diabetes mellitus | No available data |
| 3 | Relationship Between Insulin Resistance Indicators and Type 2 Diabetes Mellitus in Romania | non-cohort study |
| 4 | Association of METS-IR index with Type 2 Diabetes: A cross-sectional analysis of national health and nutrition examination survey data from 2009 to 2018 | non-cohort study |
| 5 | Association between METS-IR and Prediabetes or Type 2 Diabetes Mellitus among Elderly Subjects in China: A Large-Scale Population-Based Study | non-cohort study |
| 6 | Assessment of first-trimester insulin resistance indices for gestational diabetes mellitus: a prospective cohort study | Not the target outcome |
| 7 | Association Analysis of Insulin Resistance Metabolic Score (METS-IR) and Gestational Diabetes Mellitus: Based on National Health and Nutrition Examination Survey Database From 2007 to 2018 | Not the target outcome |
| 8 | Association between the cumulative metabolic score for insulin resistance and diabetes mellitus incidence: a prospective nationwide cohort study in China | Not the target outcome |
| 9 | Predicting incident type 2 diabetes in a Japanese cohort: an 8-year analysis of the NAGALA database | lack of specific METS-IR values |
| 10 | Metabolic score for insulin resistance (METS-IR) as a predictor of gestational diabetes: Findings from a prospective Iranian cohort study | Not the target outcome |
| 11 | Association of metabolic score for insulin resistance with gestational diabetes mellitus: a multicenter cohort study | Not the target outcome |
| 12 | Association Analysis of Insulin Resistance Metabolic Score (METS-IR) and Gestational Diabetes Mellitus: Based on National Health and Nutrition Examination Survey Database From 2007 to 2018 | Not the target outcome |
| 13 | The Role of METS-IR in Early Screening for Gestational Diabetes Mellitus in Chinese Women: A Two-Center Prospective Study | Not the target outcome |
| 14 | Non-insulin-based markers of insulin resistance at diabetes diagnosis: A pooled analysis of 14 national health surveys | Not the target outcome |
| 15 | Association of metabolic score for insulin resistance with progression or regression of prediabetes: evidence from a multicenter Chinese medical examination cohort study | Not the target outcome |
| 16 | Assessing the validity of METS-IR for predicting the future onset of diabetes: an analysis using time-dependent receiver operating characteristics | Not the target outcome |
| 17 | Association between MetS-IR and prediabetes risk and sex differences: a cohort study based on the Chinese population | Not the target outcome |
| 18 | A nonlinear associations of metabolic score for insulin resistance index with incident diabetes: A retrospective Chinese cohort study | Not the target outcome |
| 19 | Associations between four insulin resistance (IR) surrogates and the risk of small cell lung cancer (SCLC) | No available data |
| 20 | Association of non-insulin-based insulin resistance indices, mean platelet volume and prostate cancer: a cross-sectional study | non-cohort study |
| 21 | Which Surrogate Marker of Insulin Resistance Among Those Proposed in the Literature Better Predicts the Presence of Non-Metastatic Bladder Cancer? | non-cohort study |
| 22 | Association between insulin resistance and breast cancer risk in perimenopausal and postmenopausal women: mediating effects of oxidative stress and inflammatory biomarkers | non-cohort study |
| 23 | Association between the metabolic score for insulin resistance and prostate cancer: a cross-sectional study in Xinjiang | non-cohort study |
| 24 | The Relationship Between Surrogate Markers of Insulin Resistance and Occurrence of Colorectal Adenoma in Individuals under 50 Years Old: A Single Center Retrospective Cross-Sectional Study | non-cohort study |
| 25 | Is Insulin Resistance a High-Risk Factor for Postmenopausal Endometrial Cancer: Insights from the Triglyceride Glucose (TyG) Index and the Metabolic Score for Insulin Resistance (METS-IR) | non-cohort study |
| 26 | The Relationship between Surrogate Markers of Insulin Resistance and Occurrence of Colorectal Adenoma in Individuals under 50 Years Old: A Single-Center Retrospective Cross-Sectional Study | non-cohort study |
| 27 | Does insulin resistance predict prostate cancer? Results from the Reduction by Dutasteride of Prostate Cancer (REDUCE) Trial | lack of specific METS-IR values |
| 28 | Diagnostic significance and predictive efficiency of metabolic risk score for fertility-sparing treatment in patients with atypical endometrial hyperplasia and early endometrial carcinoma | lack of specific METS-IR values |
| 29 | Association between Surrogate Markers for Insulin Resistance and Incidence of Colorectal Cancer in Korea: A Nationwide Population-Based Study | No available data |
| 30 | Insulin Resistance Indices Predict Mortality in Cardiovascular Disease: A Large-Scale NHANES Study With Machine Learning Validation | No available data |
| 31 | Association of different insulin resistance surrogates with all-cause and cardiovascular mortality among the population with cardiometabolic multimorbidity | No available data |
| 32 | Sex-specific differences in the prognostic value of METS-IR for long-term outcomes in patients with MASLD and advanced liver fibrosis: a nationwide study | No available data |
| 33 | The association between novel metabolic parameters and all-cause/cardiovascular mortality in patients with metabolic syndrome is modified by age | No available data |
| 34 | Metabolic score for insulin resistance (METS-IR) predicts all-cause and cardiovascular mortality in the general population: evidence from NHANES 2001-2018 | No available data |
| 35 | Association between different insulin resistance indices and all-cause mortality in patients with diabetic kidney disease: a prospective cohort study | No available data |
| 36 | Association of the metabolic score for insulin resistance with cardiovascular diseases, cardiovascular and all-cause mortality in Chinese hypertensive population | No available data |
| 37 | Association of cardiometabolic factors and insulin resistance surrogates with mortality in participants from the Korean Genome and Epidemiology Study | No available data |
| 38 | Association between triglyceride glucose-body mass index with all-cause and cardiovascular mortality in adults with osteoporosis: a prospective study | No available data |
| 39 | Predictive value of obesity-related indices for incident type 2 diabetes mellitus: a longitudinal study of the Fukushima Health  Database 2015–2021 | No available data |

**Supplementary Table S4** Basic characteristics of the studies included in the review

|  | Author (year) | Country | Study design | Study population | Sample size | age (years) | Male (%) | Follow-up years | Mets-IR analysis | Outcomes reported | Adjustments |
| --- | --- | --- | --- | --- | --- | --- | --- | --- | --- | --- | --- |
| 1 | Huang et al.,2026 | China, Japan | RC | Adults without diabetes | Chinese: 114,293 ; Japanese : 15,453 | Chinese: 44.01; Japanese: 43.71 | Chinese: 53.15% ; Japanese : 54.48% | Chinese : 3.10; Japanese : 6.05 | continuous | Incident type 2 diabetes | age, sex, systolic blood pressure, diastolic blood pressure, low-density lipoprotein cholesterol, alanine aminotransferase, aspartate aminotransferase, blood urea nitrogen, serum creatinine, smoking status, alcohol consumption, and family history of diabetes |
| 2 | Hu et al.,2025 | Britain | PC | UK Biobank participants aged over 40 years | 312,380 | 55.63 | 45.14% | 13.84 | continuous categorical | Incident type 2 diabetes；all-cause mortality | Age, sex, ethnicity,education level, annual household income, BMI group, mean blood pressure (MBP), smoking/drinking status, serum uric acid, estimated glomerular filtration rate (eGFR), low-density lipoprotein cholesterol (LDL-C) |
| 3 | Cheng et al.,2024 | China | PC | Chinese middle-aged and older adults aged ≥45 years, free of T2DM at baseline | 7583 | 59.1 | 46.60% | 6.3 | continuous categorical | Incident type 2 diabetes | Adjusted for demographics (age, sex, place of residence, education level), socioeconomic status (annual household income, living relationships), lifestyles (cigarette smoking, alcohol drinking), and anthropometric/clinical parameters (blood pressure [BP], waist circumference) |
| 4 | Zhang et al.,2021 | China | PC | Residents from rural areas in Luoyang City, Henan Province, China | 12,107 | 50.48 | 37.91% | 5.93 | continuous categorical | Incident type 2 diabetes | Sex, age, educational level, marital status, family history of diabetes, smoking, alcohol drinking, physical activity，waist circumference (WC), systolic blood pressure (SBP), total cholesterol (TC) (baseline METS-IR was additionally adjusted when analyzing the association between absolute METS-IR change and incident T2DM) |
| 5 | Cai et al.,2021 | Japan | PC | Non-obese Japanese adults | 12,290 | 43.43 | 52.25% | 5.6 | continuous categorical | Incident type 2 diabetes | Age, sex, waist circumference (WC)，regular exercise, smoking status, alcohol consumption，fatty liver, haemoglobin A1c (HbA1c)，systolic blood pressure (SBP), alanine aminotransferase (ALT), aspartate aminotransferase (AST), gamma-glutamyl transferase (GGT), high-density lipoprotein cholesterol (HDL-C), total cholesterol (TC), triglycerides (TG) |
| 6 | Bello-Chavolla et al.,2018 | Mexico | PC | Mexican adults | 6,144 | 42.63 | 33.40% | 2.42 | Continuous  categorical | Incident type 2 diabetes | age, sex, family history of T2DM, hypertension, physical activity, waist circumference, and smoking |
| 7 | Shine et al.,2025 | Korea | PC | Korean adults who underwent national health screening in 2010–2011 | 322,624 | 58.7 | 53.90% | 9.5 | categorical | Incidence of lung cancer (ICD-10 codes C33–C34) | age, sex, income level, residential area, hypertension, diabetes, dyslipidemia, Charlson Comorbidity Index (CCI), hemoglobin, estimated glomerular filtration rate (eGFR), smoking, alcohol consumption, and regular exercise |
| 8 | Yang et al.,2024 | Britain | PC | UK adults aged 37–73 years | 388,900 | 57 | 47.20% | 13 | continuous categorical | Incidence of esophageal cancer | age， sex，ethnicity, Townsend deprivation index, physical activity (MET), smoking/alcohol status, diabetes mellitus, hypertension, insulin use, fasting time, and diet score |
| 9 | Wang et al.,2024 | Britain | PC | UK adults aged 40–69 years | 395,304 | 56.3 | 46.40% | 11.03 | continuous；categorical | Incidence of lung cancer (ICD-10 codes C33–C34) | age, sex, BMI, Townsend deprivation index, education level, smoking status, pack-years of smoking, alcohol intake frequency, ethnicity, and diabetes |
| 10 | Son et al.,2024 | Korea | PC | Korean adults aged ≥40 years who underwent national health screening in 2009–2010 | 314,141 | 58.8 | 53.90% | 10 | categorical | Incidence of colorectal cancer (CRC, ICD-10 codes C18–C20) | age, sex, income level, residential area, hypertension, diabetes, dyslipidemia, Charlson Comorbidity Index (CCI), hemoglobin, estimated glomerular filtration rate (eGFR), smoking, alcohol consumption, and regular exercise |
| 11 | Kim et al.,2024 | Korea | PC | Korean adults aged ≥40 years who underwent national health screening in 2009–2010 | 314,321 | 58.8 | 53.90% | 9.5 | categorical | Incidence of thyroid cancer (TC, ICD-10 code C73) | age, sex, income level, residential area, hypertension, diabetes, dyslipidemia, Charlson Comorbidity Index (CCI), hemoglobin, estimated glomerular filtration rate (eGFR), smoking, alcohol consumption, and regular exercise |
| 12 | Wang et al.,2026 | American | PC | U.S. adults aged ≥20 years with cardiovascular–kidney–metabolic (CKM) syndrome stages 1–4 | 7,741 | 52 | 57.00% | 8.3 | continuous categorical | All-cause mortality | demographic variables (age, gender, ethnicity, education, marital status, poverty income ratio, smoking, alcohol consumption, physical activity)，laboratory indices (ALT, albumin, UACR, AST, TC, eGFR, HbA1c), vital signs (SBP, DBP), anthropometric measures (BMI, waist), and comorbidities (hypertension, diabetes, coronary heart disease, stroke, metabolic syndrome) |
| 13 | Zhu et al.,2025 | American | PC | US adults with diabetic kidney disease (DKD) | 1588 | 64.64 | 50.97% | 6.2 | continuous categorical | All-cause mortality | age, gender, ethnicity，education level, income-to-poverty ratio, BMI, liver/kidney function indices, lipids, serum electrolytes, hypertension, and cardiovascular disease |
| 14 | Yang et al.,2025 | American | PC | US elderly adults aged ≥65 years with diabetes | 1248 | 72 | 53.90% | 6.1 | continuous | All-cause mortality | sociodemographic, clinical, laboratory factors, comorbidities, and medication use |
| 15 | Liu et al.,2025 | American | PC | US adults with osteoporosis or osteopenia | 2175 | 56.04 | 49.33% | 16 | continuous | All-cause mortality | sex, age, race，education, poverty-to-income ratio, hypertension, diabetes, alcohol use, smoking status, physical activity level |
| 16 | Su et al.,2024 | American | PC | Adults with cardiovascular disease (CVD) | 2,515 | 64 | 54.61% | 7.6 | continuous categorical | All-cause mortality | age, gender, and race，education level, smoking status, marital status, alcohol drinking, BMI, waist circumference, poverty income ratio (PIR), physical activity (PA), LDL-cholesterol, total cholesterol (TC), HbA1c, estimated glomerular filtration rate (eGFR), hypertension, and diabetes status |
| 17 | Li et al.,2024 | American | PC | US adults aged ≥20 years | 19,204 | 47.12 | 49.31% | 9.17 | continuous categorical | All-cause mortality | age, gender, race/ethnicity, education level, marital status, smoking, alcohol consumption, BMI, Healthy Eating Index (HEI)-2015, and physical activity，hypertension, hypercholesterolemia, and cancer history |
| 18 | Wang et al.,2022 | American | PC | U.S. adults aged ≥18 years with diabetes | 2,542 | 57.12 | 52.80% | 7.5 | continuous categorical | All-cause mortality | age, gender, and race/ethnicity，BMI, education level, family income-poverty ratio, alcohol use, smoking status, ideal physical activity, and daily calorie intake，diabetes duration, hypoglycemic/lipid-lowering drugs, self-reported hypertension, hypercholesterolemia, CVD history, systolic blood pressure, and total cholesterol |
| 19 | Ryu et al.,2024 | Korea | PC | Korean adults aged ≥60 years | 30,164 | 64.3 | 42.60% | 11.7 | categorical | All-cause mortality | age, sex, and waist circumference (WC)，systolic blood pressure (SBP), diastolic blood pressure (DBP), alanine aminotransferase (ALT), aspartate aminotransferase (AST), creatinine, and low-density lipoprotein (LDL) cholesterol，smoking status, alcohol intake, exercise, hypertension, and type 2 diabetes |
| 20 | Zhang et al.,2024 | China | PC | Chinese hypertensive adults aged ≥18 years | 14,220 | 63.8 | 47.20% | 3.94 | continuous categorical | All-cause mortality | age, gender, and BMI，systolic blood pressure, diastolic blood pressure, AST, ALT, antihypertensive drugs, lipid-lowering drugs, hypoglycemic drugs, and current smoking status |
| 21 | Xing et al.,2025 | American | PC | US nondiabetic adults aged 45–64 years at baseline (1987–1989) | 7835 | 53.9 | 44.70% | 23.3 | categorical | All-cause mortality | age, race, education, research center， smoking, alcohol consumption, BMI, family history of CVD, blood pressure, antihypertensive/lipid-lowering medications, fasting glucose, estimated glomerular filtration rate (eGFR) |
| 22 | Zhou et al.,2024 | China | RC | Patients with heart failure with preserved ejection fraction (HFpEF) aged ≥18 years | 3,248 | 65.7 | 59.00% | 4.15 | continuous categorical | All-cause mortality | age, gender, heart rate, New York Heart Association (NYHA) classification, and left ventricular ejection fraction (LVEF),T-proBNP, hemoglobin, creatinine, low-density lipoprotein cholesterol atrial fibrillation, hypertension, stroke, peripheral arterial disease (PAD), ischemic etiology, statins, ACEI/ARB/ARNI, beta-blockers, calcium channel blockers (CCB), mineralocorticoid antagonists, diuretics, insulin, and SGLT2 inhibitors |
| 23 | Zadeh et al.,2024 | Iran | PC | Iranian adults aged ≥20 years | 12,790 | 41.7 | 43.10% | 17.8–17.9 | continuous categorical | All-cause mortality | age and gender，current smoking, diabetes/prediabetes, hypertension/elevated BP, prevalent CVD/family history of premature CVD, family history of diabetes (for diabetes outcome), non-HDL-C, lipid-lowering drug use, pulse rate, and serum creatinine，metabolic syndrome (METS) |
| PC:Prospective cohort study;RC:Retrospective cohort study; | | | | | | | | | | | |

**Supplementary Table S5** Quality assessment of studies included in the review

| Study | Representativeness of the exposed cohort | Selection of the eon-exposed cohort | Ascertainment of exposure | Outcome not present at baseline | Comparability of cohorts on the basis of the design or analysis | Assessment of outcome | Sufficient follow-up duration | Adequacy of follow up of eohorts | Overall |
| --- | --- | --- | --- | --- | --- | --- | --- | --- | --- |
| Huang et al.,2026 | 1 | 1 | 1 | 1 | 2 | 1 | 1 | 1 | 9 |
| Hu et al.,2025 | 1 | 1 | 1 | 1 | 2 | 1 | 1 | 1 | 9 |
| Cheng et al.,2024 | 1 | 1 | 1 | 1 | 2 | 1 | 1 | 0 | 8 |
| Zhang et al.,2021 | 1 | 1 | 1 | 1 | 2 | 1 | 1 | 1 | 9 |
| Cai et al.,2021 | 1 | 1 | 1 | 1 | 2 | 1 | 1 | 1 | 9 |
| Bello-Chavolla et al.,2018 | 1 | 1 | 1 | 1 | 2 | 1 | 1 | 0 | 8 |
| Shine et al.,2025 | 1 | 1 | 1 | 1 | 2 | 1 | 1 | 1 | 9 |
| Yang et al.,2024 | 1 | 1 | 1 | 1 | 2 | 1 | 1 | 1 | 9 |
| Wang et al.,2024 | 1 | 1 | 1 | 1 | 2 | 1 | 1 | 1 | 9 |
| Son et al.,2024 | 1 | 1 | 1 | 1 | 2 | 1 | 1 | 0 | 8 |
| Kim et al.,2024 | 1 | 1 | 1 | 1 | 2 | 1 | 1 | 1 | 9 |
| Wang et al.,2026 | 1 | 1 | 1 | 1 | 2 | 1 | 1 | 0 | 8 |
| Zhu et al.,2025 | 1 | 0 | 1 | 1 | 2 | 1 | 1 | 1 | 8 |
| Yang et al.,2025 | 1 | 1 | 1 | 1 | 2 | 1 | 1 | 0 | 8 |
| Liu et al.,2025 | 1 | 1 | 1 | 1 | 2 | 1 | 1 | 0 | 8 |
| Su et al.,2024 | 1 | 0 | 1 | 1 | 2 | 1 | 1 | 1 | 8 |
| Li et al.,2024 | 1 | 1 | 1 | 1 | 2 | 1 | 1 | 0 | 8 |
| Wang et al.,2022 | 1 | 1 | 1 | 0 | 2 | 1 | 1 | 1 | 8 |
| Ryu et al.,2024 | 1 | 1 | 1 | 1 | 2 | 1 | 1 | 0 | 8 |
| Zhang et al.,2024 | 1 | 1 | 1 | 1 | 2 | 1 | 1 | 1 | 9 |
| Xing et al.,2025 | 1 | 1 | 1 | 1 | 2 | 1 | 1 | 0 | 8 |
| Zhou et al.,2024 | 1 | 1 | 1 | 1 | 2 | 1 | 1 | 0 | 8 |
| Zadeh et al.,2024 | 1 | 1 | 1 | 1 | 2 | 1 | 1 | 1 | 9 |

**Supplementary Fig. 1** METS-IR (highest vs. lowest) and subgroup analysis of cancer

**Supplementary Fig. 2** METS-IR (highest vs. lowest) and subgroup analysis of all-cause mortality

**Supplementary Fig. 3** Subgroup Analysis of METS-IR (per SD) and All-Cause Mortality

**Supplementary Fig. 4** Sensitivity analysis

**Supplementary Fig. 5** Analysis results of publication bias
